# The application of brain-computer interface on cognitive function and activities of daily living in stroke patients: a systematic review and meta-analysis

**DOI:** 10.3389/fneur.2026.1862193

**Published:** 2026-10-19

**Authors:** Zijie Luo, Lei Yu, Meiling Wu, Xuan Gong

**Affiliations:** 1 Department of Neurological Rehabilitation, Zhongnan Hospital of Wuhan University, Wuhan, Hubei, China; 2 Rehabilitation Medicine Center, Hubei Provincial Hospital of Integrated Traditional Chinese and Western Medicine, Wuhan, Hubei, China

**Keywords:** activities of daily living, brain–computer interface, cognitive function, meta-analysis, stroke

## Abstract

**Objective:**

Nonmotor dysfunction is a common sequela of stroke, affecting patients across acute, subacute, and chronic recovery stages and significantly impairing their functional independence, psychosocial adjustment, and overall Activities of Daily Living (ADL). Brain-computer interface (BCI) technology has demonstrated effectiveness in motor function rehabilitation. The primary objective of this study was to assess the efficacy of BCI technology on cognitive function (as the primary indicator of nonmotor dysfunction) and ADL in stroke patients through a systematic review and meta-analysis.

**Methods:**

This review was conducted in accordance with the PRISMA guidelines and registered with PROSPERO (CRD420261453809). Systematic searches were conducted in PubMed, Web of Science, Google Scholar, CNKI, and Wanfang Data up to March 2026. Statistical analyses and the risk of bias were assessed using Review Manager 5.3. The quantitative synthesis focused on the effects of BCI on the cognitive function and ADL of patients with stroke.

**Results:**

The meta-analysis reviewed 11 randomized controlled studies (RCTs) involving a total of 559 participants. Compared with conventional rehabilitation, BCI-based training significantly improved cognitive function (SMD = 0.634, 95% CI: 0.214, 1.055; *p* = 0.003) and ADL (SMD = 1.198, 95% CI: 0.079, 2.316; *p* = 0.036). Subgroup analyses suggested exploratory patterns of benefit in certain strata. However, subgroup analysis revealed no significant differences for most comparisons (including scale type, region, sample size, stroke type, BCI type, intervention intensity, and intervention duration; all *p* > 0.05), except for first-episode stroke in the ADL analysis (*p* = 0.001).

**Conclusion:**

BCI-based training shows preliminary promise for improving cognitive function and ADL in stroke patients. Current evidence remains insufficient to recommend BCI for emotion or speech rehabilitation. However, the GRADE certainty of evidence was rated as low for cognitive function and very low for ADL, indicating that further high-quality research is warranted.

**Systematic review registration:**

https://www.crd.york.ac.uk/prospero/, CRD420261453809.

## 1 Introduction

Stroke has emerged as the leading cause of long-term adult disability worldwide and one of the most fatal diseases in the last few decades, placing substantial economic and social burdens on health systems, families, and societies globally (1). According to data from the World Health Organization, the number of disability-adjusted life years caused by stroke increased from 137 million in 2000 to 160 million in 2021, indicating that stroke poses a significant challenge to public health (1). For a long time, clinical research and rehabilitation practices for stroke have focused primarily on motor dysfunctions such as hemiplegia, limb spasticity, and gait abnormalities. However, growing evidence suggests that nonmotor dysfunctions poststroke have a greater effect on Activities of Daily Living (ADL) than motor impairments do (2). These nonmotor dysfunctions can persist through the acute, subacute, and chronic recovery phases, profoundly affecting patients’ functional independence, psychosocial adaptation, and overall ADL. However, these dysfunctions are often overlooked in clinical settings (3).

Nonmotor dysfunctions after stroke include mainly cognitive impairment, emotional disorders, sleep disorders, fatigue, speech disorders, swallowing dysfunction, poststroke central pain, and abnormalities in urinary and bowel functions (4). Ozkan et al. (5) reported that nearly 90% of patients experienced impairment in at least one nonmotor dysfunction domain within 30 days after acute stroke, and 50% of patients had three or more concurrent nonmotor impairments. Further studies confirmed that 6 months post-stroke, 75% of patients still had at least one affected nonmotor dysfunction domain. Fatigue, sleep disorders, and reduced social participation impact more than 50% of survivors, with 26% of patients reporting more than five adverse outcomes (6). These impairments are not secondary manifestations of motor dysfunction but are independent predictors of poor rehabilitation outcomes, reduced socio-occupational participation, prolonged hospitalization, increased readmission rates, and even increased mortality. Therefore, improving nonmotor functions and enhancing patients’ ADL, in addition to motor function recovery, have become core clinical goals in stroke rehabilitation.

Current interventions for poststroke nonmotor dysfunction primarily include pharmacological therapy, cognitive behavioral therapy, speech therapy, and supportive care. While these methods may achieve certain efficacy in some patients, they have significant clinical limitations. For example, drug treatments have adverse effects and drug interactions, along with poor long-term compliance, which is particularly evident in elderly patients with multiple underlying conditions (4). Additionally, cognitive rehabilitation interventions require substantial human resources and rely heavily on patient cooperation and therapist expertise, and their efficacy often diminishes gradually in patients with severe or chronic nonmotor dysfunction (7). Furthermore, conventional rehabilitation (CR), such as traditional cognitive rehabilitation, does not significantly improve overall cognitive function, and single-attention training provides only short-term benefits without lasting enhancement in cognitive function or ADL (8). Moreover, traditional rehabilitation programs lack targeted psychological intervention modules, making it difficult to effectively address poststroke depression (9). Moreover, conventional treatments lack robust evidence for alleviating poststroke fatigue and fail to effectively address the multifactorial pathological mechanisms (neurological, immune, psychological, etc.) of poststroke fatigue (10). Therefore, there is an urgent clinical need to explore novel, targeted, and precisely adjustable rehabilitation technologies to improve nonmotor function and ADL in stroke patients.

Brain–computer interface (BCI) technology, as a disruptive neurorehabilitation method, is a system that translates or decodes brain activity signals into instructions, enabling direct control of external devices without relying on peripheral nerves or muscles, and has been widely applied in motor function rehabilitation (11). Signal acquisition methods can be broadly categorized into invasive and noninvasive types. Among these, electroencephalogram-based systems (EEG-BCIs) have gradually been used for interventions targeting nonmotor dysfunctions in recent years because of their advantages of high safety, ease of operation, and real-time neural feedback (12). Current research has applied BCI to cognitive enhancement, emotion regulation, speech rehabilitation, and sleep improvement in stroke patients and has confirmed the positive effects of BCI on neurobehavioral functions and ADL (13). However, clinical evidence for the use of BCI in the rehabilitation of nonmotor dysfunctions poststroke remains inadequate. Existing studies generally suffer from small sample sizes, inconsistent intervention protocols, diverse outcome measures, significant variations in control designs, and high heterogeneity across studies. Moreover, few studies have comprehensively summarized the efficacy of BCIs on nonmotor functions and ADL in stroke patients.

This study systematically searches, evaluates, and quantitatively integrates all clinical trials that compare BCI interventions with CR or standard care. In this study, the modified Barthel index (MBI), an outcome measure for assessing ADL, was used as a secondary outcome. We evaluated the comprehensive effect size of BCIs on patients’ nonmotor functions and subjective ADL and explored the sources of heterogeneity in the studies, including patients’ baseline characteristics, BCI types, intervention duration, and training protocols. This study clarifies the clinical value of BCI in nonmotor rehabilitation for stroke patients by providing a comprehensive quantitative synthesis of the current evidence, hoping to offer a basis for developing rehabilitation guidelines, and identifies future clinical research priorities, aiming to promote the integration of BCI technology into stroke rehabilitation systems, alleviate the burden of nonmotor symptoms in patients, facilitate functional recovery, and improve long-term ADL for stroke survivors. Notably, owing to the limited number of available studies on emotion, speech, swallowing, and other nonmotor domains, the quantitative synthesis in this meta-analysis focused primarily on cognitive function as the core indicator of nonmotor outcomes. Other nonmotor domains are discussed qualitatively.

## 2 Methods

### 2.1 Search strategy

The following electronic databases were searched from their inception to March 2026: PubMed, Web of Science, Google Scholar, WanFang, and CNKI. The search was limited to studies published in English or Chinese. Search keywords: (Brain-Computer Interface OR BCI OR Brain Computer Interface) AND (Stroke OR Cerebral Infarction OR Cerebral Hemorrhage) AND (Nonmotor Function OR Cognition OR Emotion OR Dysphagia OR Speech Disorder OR Balance OR Sensation).

Protocol Registration: This systematic review protocol was retrospectively registered with PROSPERO on 17 July 2026 (CRD420261453809). The PRISMA 2020 checklist is provided as a Supplementary file.

### 2.2 Inclusion and exclusion criteria

Inclusion criteria:

1) Participants: Stroke patients.
2) Interventions: BCI (invasive/noninvasive, including combined feedback/stimulation).
3) Study type: Randomized controlled trials (RCTs).
4) Outcome measures: Primary outcome: This outcome is reported as nonmotor functions (this study focuses on cognitive function as the core evaluation indicator of nonmotor functions, primarily using Montreal Cognitive Assessment (MoCA) data for meta-analysis; if studies also report other cognitive scales such as the Mini-Mental State Examination (MMSE), these can be combined as supplementary indicators for cognitive function. Other nonmotor functions, such as mood, speech, and swallowing, were not quantitatively synthesized because the number of included studies was insufficient;
5) Secondary outcomes: Reporting ADL.
6) Accessibility: Full-text articles provided.

Exclusion criteria:

1) Duplicate publications or incomplete data;
2) Missing clinical data;
3) Nonclinical studies (animal experiments, *in vitro* experiments, reviews, and conference abstracts), case reports, case series, cross-sectional studies, and other uncontrolled observational studies.

For the purpose of this review, BCI was defined as a system that acquires central nervous system signals (e.g., EEG), translates them in real time into artificial outputs via signal processing and classification algorithms, and thereby provides users with communication or control capabilities that do not depend on peripheral nerves or muscles (14). Accordingly, we included studies employing EEG-based BCI systems, encompassing both motor imagery-based BCIs and attention-monitoring neurofeedback systems.

### 2.3 Data collection and processing

#### 2.3.1 Data extraction from published articles and reports

Two independent reviewers screened the titles, abstracts, and full texts according to the inclusion criteria. If there was a disagreement between the two researchers, a third researcher arbitrated. Data were collected from the included studies by two independent researchers using a standardized data sheet containing the following items: first author, year of publication, region, study design, sample size, age, sex, nonmotor function scale, and ADL scale. For each included study, we attempted to extract the technical specifications of the BCI (e.g., signal acquisition, feature extraction, and classification algorithms). However, most primary studies did not report sufficient technical details in these areas. The available information is summarized in Supplementary Table S1 for transparency.

#### 2.3.2 Study risk of bias or quality assessment

Methodological quality of the included RCTs was assessed using the Cochrane Risk of Bias 2 (RoB 2) tool, implemented via Review Manager 5.4.1. Data were assessed by at least two independent individuals (or a combination of individuals/machines), and a discrepancy resolution process was established. If the required information in the study publication/report was unclear or unavailable, the researchers were contacted to request Supplementary materials.

#### 2.3.3 Assessment of reporting bias

Two authors conducted the risk of bias assessment. If necessary, a third author resolved disagreements between the two authors to reach a consensus.

#### 2.3.4 Certainty assessment

The GRADE approach was used to assess the certainty of evidence for each outcome, which was rated as high, moderate, low, or very low based on five domains: risk of bias, inconsistency, indirectness, imprecision, and publication bias. Two independent reviewers performed the assessment, with disagreements resolved by a third reviewer.

### 2.4 Planned data synthesis

#### 2.4.1 Strategy for data synthesis

Meta-analyses, including forest plot generation, sensitivity analysis, and subgroup analysis, were performed using Review Manager 5.3, with funnel plots and publication bias tests (Begg’s and Egger’s tests) performed using Stata 18.0. The Cochrane Q statistic was used to test heterogeneity among studies, and the degree of heterogeneity was assessed using the I^2^ statistic: I^2^ values of 25, 50, and 75% corresponded to low, moderate, and high heterogeneity, respectively. When significant heterogeneity was detected by the Cochrane Q statistic, a random-effects model was employed to calculate the pooled prevalence and 95% confidence interval (CI); otherwise, a fixed-effects model was used. The statistical significance threshold was set at *p* < 0.05. The study results are presented in forest plots.

Since different cognitive tools (MoCA, SDMT, and LOCTA) and ADL scales were used across studies, we calculated the standardized mean difference (SMD) as the effect size metric to standardize the results to a common scale in accordance with the Cochrane Handbook recommendations.

Given the clinical heterogeneity expected across studies (e.g., diverse BCI protocols, patient populations, and control interventions), we applied the DerSimonian–Laird random-effects model for all pooled estimates. To account for the use of different cognitive and ADL instruments across studies, we used the standardized mean difference (SMD) as the effect size metric. All extracted data were independently verified against the original publications, and any unit errors were corrected prior to analysis.

#### 2.4.2 Analysis of subgroups or subsets

Subgroup analyses were conducted on the basis of region (Asia vs. Europe), sample size (n < 30, 30 ≤ n ≤ 50, n > 50), stroke type (mixed/unclassified vs. ischemic), intervention intensity (low: 2–3 sessions/week; moderate: 5–6 sessions/week; high: 7 sessions/week), intervention duration (≤14 days vs. ≥21 days), and whether it was the first stroke (first-ever vs. nonfirst). These subgroup factors were selected on the basis of clinical relevance and the distribution of available data. None of the subgroup analyses were pre-specified in the PROSPERO registration (CRD420261453809), and all subgroup findings are exploratory. BCI type subgroup analysis was not performed because of the conceptual overlap between EEG-based signal acquisition and MI-based control paradigms and because of the limited number of studies per category.

## 3 Results

As of March 2026, a preliminary search identified 935 publications (including 467 from PubMed, 232 from Google Scholar, 158 from Web of Science, 62 from WanFang, and 16 from CNKI). After removing duplicates, 416 articles were retained for title and abstract screening. On the basis of the inclusion and exclusion criteria, 124 articles underwent full-text review, with 11 original studies (15–25) ultimately included. Among these, 6 studies reported only nonmotor function outcomes (16, 17, 20, 22, 23, 25), 3 studies reported only ADL outcomes (18, 21, 24), and 2 studies reported both types of outcomes (15, 19) (Figure 1).

**Figure 1:**
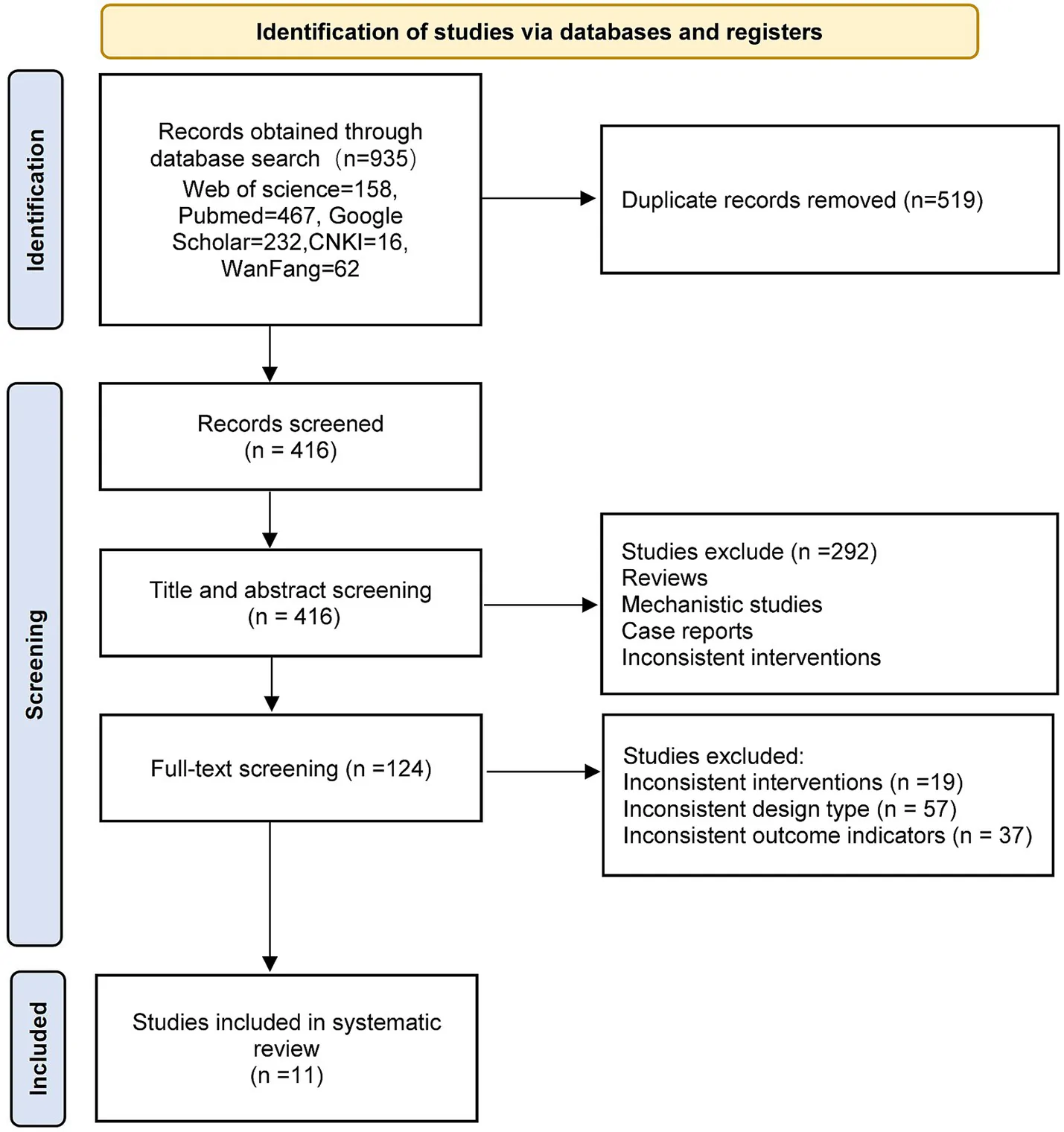
Preferred reporting items for systematic reviews and meta-analyses statement flowchart.

### 3.1 Study characteristics and quality assessment

Table 1 summarizes the baseline characteristics of the included studies, detailing participants, interventions, and outcome measures. This review included 11 RCTs, with 7 conducted in China, 2 in Russia, 1 in Iran, and 1 in Portugal. A total of 559 stroke patients participated, with an average age ranging from 37 to 86 years. The experimental interventions involved the BCI and CR, whereas the control group received corresponding treatments without the BCI component. In addition to methodological quality, we assessed the overall certainty of evidence for each outcome using the GRADE approach. A summary of the findings is presented in Table 2.

**Table 1:** Characteristics of the included studies.

First author, year of publication	Region	BCI type	BCI training intensity	Types of stroke	Sample size	BCI sample size	Control sample size	Age	Gender	Cognitive scale	Activities of daily living scale	Intervention duration (Day)	Stroke course
Liu et al. 2023 (15)	China	MI-BCI	Five times a week	Stroke	60	30	30	51.20 ± 13.87	Female: 19 31.67% male 41 68.33%	SDMT	Modified Barthel Index (MBI)	21	Less than or equal to one month
Wan et al. 2025 (16)	Chnia	MI-VR-BCI	Five times a week	Stroke	30	14	16	56.63 ± 13.42	Female: 6 20% male: 24 80%	SDMT	—	28	Within 1 to 12 months of stroke onset
Isakova et al. 2025 (17)	Russia	BFB-BCI-EEG BFB-BCI-P300	Seven times a week	Stroke	89	70	19	61.29 ± 10.68	—	MoCA	—	10	Subacute, 1–9 months after a stroke
Ming et al. 2025 (18)	China	KI-BCI BCI-tDCS	Five times a week	First-time stroke patients, ischemic stroke, hemorrhagic stroke	43	29	14	57.40 ± 8.81	Female: 16 37.21% male 27 62.79%	—	MBI	28	The inclusion criteria are for the subacute phase of stroke (more than or equal to 14 days and less than 180 days after onset).
Zhao et al. 2022 (19)	China	SSMVEP-EEG-BCI	Six times a week	Ischemic stroke, hemorrhagic stroke	28	14	14	53.10 ± 11.50	Female: 3 10.7% male 25 89.3%	LOCTA(Loewenstein Occupational Therapy Cognitive Assessment)	MBI	28	Subacute stroke, with a course of 2 weeks to 3 months after onset
Fateeva et al. 2023 (20)	Russia	EEG-BCI-P300	Seven times a week	Ischemic stroke	29	14	15	57.19 ± 6.70	Female: 10 34.48% male 19 65.52%	MoCA	—	10	Onset less than 3 months
Hao et al. 2023 (21)	China	EEG-BCI-RTS-FES	Seven times a week	Stroke	66	33	33	58.26 ± 7.69	Female: 33 50% male 33 50%	—	MBI	70	55–95 days after stroke
Yuan et al. 2021 (22)	China	BCI-PT	Six times a week	Ischemic stroke	30	16	14	63.32 ± 7.24	female 13 43.3% male 17 56.7%	SDMT	—	14	Early subacute, the inclusion criteria are within 1 week to 3 months from the onset of the first stroke
Amiri et al. 2023 (23)	Iran	Rehacom-BCI	Two times a week	Ischemic stroke	50	25	25	53.74 ± 10.81	Female: 20 40% male 30 60%	SDMT	—	35	4–12 months after stroke onset
Wang et al. 2023 (24)	China	EEG-BCI	Five times a week	First-time stroke patients, ischemic stroke, hemorrhagic stroke	120	60	60	77.74 ± 7.44	Female: 28 23.33% male 92 76.67%	—	WHOADL-BREF	180	4 weeks to 6 months
Valente et al. 2026 (25)	Portugal	EEG-VR-MI-BCI	Three times a week	Ischemic stroke, hemorrhagic stroke	14	9	5	58.00 ± 6.00	Female: 5 35% male 9 65%	MoCA	—	28	Chronic stroke (poststroke > 6 months at the start of intervention), poststroke months range from 5.4 to 181.5 months

**Table 2:** Summary of findings and GRADE certainty of evidence.

Outcome	Studies (N)	Effect (SMD, 95% CI)	Heterogeneity	GRADE	Reasons for = downgrading
Non-motor function	8 (330)	0.634 (0.214, 1.055)	*I*^2^ = 66.3%, *p* = 0.004	Low ⨁⨁◯◯	Risk of bias (performance bias due to inability to blind participants/personnel); Inconsistency (substantial unexplained heterogeneity)
Activities of daily living	5 (317)	1.198 (0.079, 2.316)	*I*^2^ = 94.5%, *p* < 0.0001	Very low ⨁◯◯◯	Risk of bias (performance bias); Serious inconsistency (very high heterogeneity); Imprecision (wide confidence interval)

### 3.2 Impact on nonmotor function and activities of daily living

#### 3.2.1 Impact on non-motor functions

A total of 8 related studies (15–17, 19, 20, 22, 23, 25) involving 330 participants demonstrated the effects of BCI-based training on nonmotor functions. The results revealed that the intervention effect of the BCI group was significantly greater than that of the CR group (SMD = 0.63, 95% CI: 0.21, 1.04). Heterogeneity analysis revealed moderate heterogeneity among the included studies (*I*^2^ = 65%, *p* = 0.006) (Figure 2A). To assess the influence of individual studies on the overall pooled effect size, sensitivity analysis was further conducted. After each study was sequentially excluded, the corresponding SMD values did not significantly change in direction or significance, indicating high robustness. Specifically, excluding Amiri et al. (23), which carried the largest effect weight (SMD = 1.683, 13.3%) and used computer-assisted cognitive training rather than EEG-based BCI, yielded a pooled SMD of 0.50 (95% CI: 0.15 to 0.84), with the significance and direction of the overall estimate unchanged (Figure 2B). The GRADE certainty of evidence for this outcome was rated as low (Table 2).

**Figure 2:**
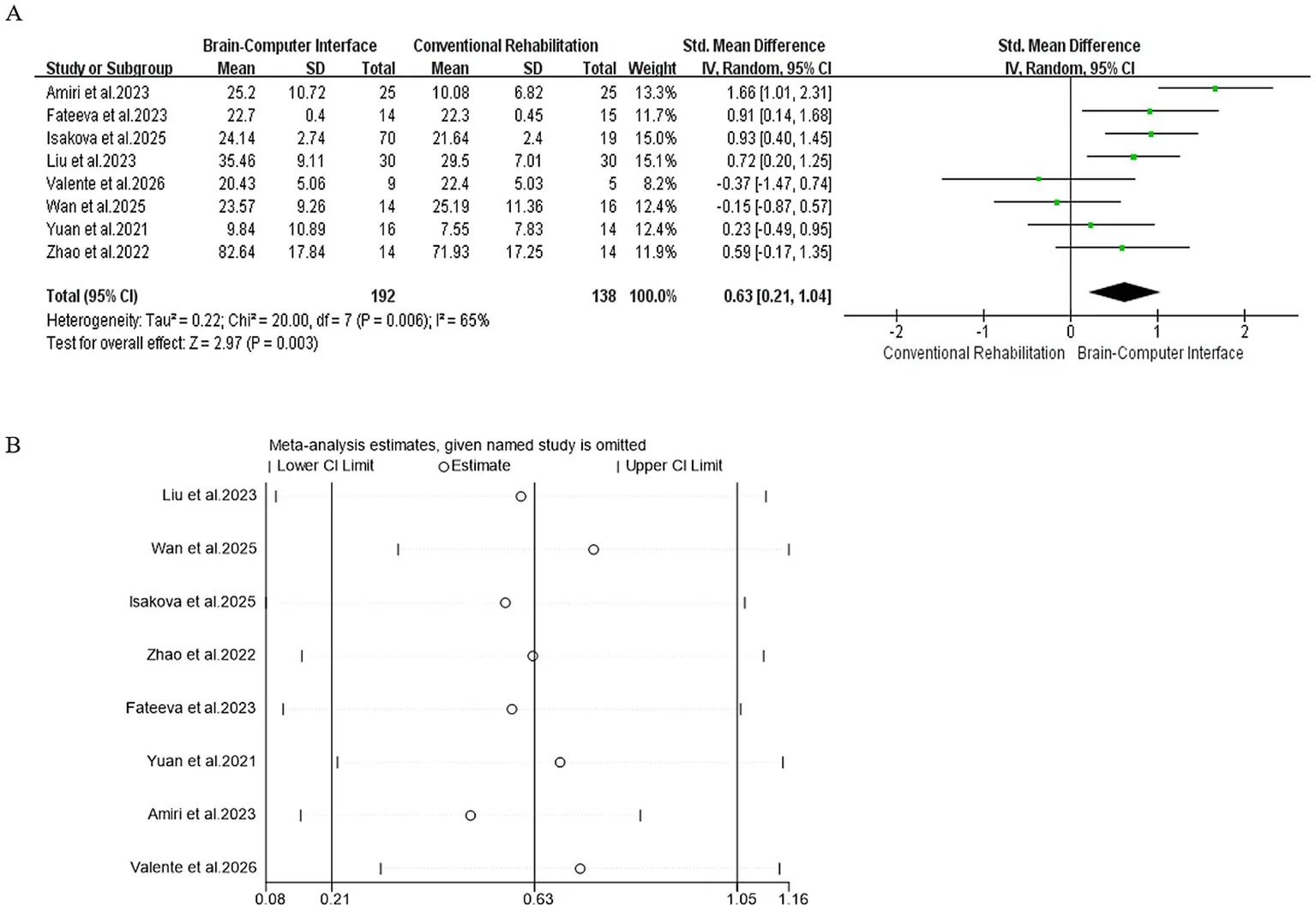
Forest plot **(A)** and sensitivity analysis plot **(B)** of the efficacy of the BCI on nonmotor dysfunction in stroke patients.

#### 3.2.2 Subgroup analysis of nonmotor functions

To evaluate the robustness of the pooled results, a series of subgroup analyses were performed on the basis of region, sample size, stroke type, BCI type, intervention intensity, and intervention duration (Table 3).

**Table 3:** Subgroup analysis of the effect of BCI efficacy on non-motor function.

Grouping	Number of Studies	Model	SMD	95% CI	I2	PH	Z	P	Heterogeneity between groups
Region									
Asia	5	Random	0.63	0.04,1.21	74.00%	0.004	2.100	0.040	1.00
Europe	3	Random	0.63	−0.04,1.30	56.00%	0.100	1.840	0.070	
Sample									
n<30	3	Random	0.48	−0.18,1.14	43.00%	0.170	1.430	0.150	0.65
30 ≤ n ≤ 50	3	Random	0.59	−0.52,1.70	87.00%	0.0005	1.040	0.300	
n>50	2	Random	0.82	0.45,1.20	0.00%	0.590	4.360	<0.0001	
Stroke									
Mixed/unclassified stroke	5	Random	0.46	0.00,0.91	54.00%	0.070	1.970	0.050	0.31
Ischemic stroke	3	Random	0.95	0.11,1.78	76.00%	0.020	2.210	0.030	
BCI training intensity									
Low intensity	2	Random	0.70	−1.28,2.68	90.00%	0.002	0.690	0.490	0.21
Moderate intensity	4	Random	0.39	−0.005,0.78	28.00%	0.240	1.940	0.050	
High intensity	2	Random	0.92	0.49,1.36	0.00%	0.996	4.225	<0.0001	
Day									
*n* ≤ 14	3	Random	0.72	0.29,1.15	23.00%	0.270	3.290	0.001	0.67
*n* ≥ 21	5	Random	0.55	−0.12,1.22	77.00%	0.002	1.620	0.110	

Subgroup analysis based on region revealed that the Asian subgroup included a total of 5 studies, with an SMD of 0.63 (95% CI: 0.04, 1.21), and high heterogeneity was detected within the subgroup (*I*^2^ = 74%, *p* = 0.004); the European subgroup included 3 studies, with an SMD of 0.63 (95% CI: −0.04, 1.30), and moderate heterogeneity was detected within the subgroup (*I*^2^ = 56%, *p* = 0.10). Intergroup heterogeneity tests revealed no statistically significant heterogeneity between the Asian and European subgroups (*p* = 1.00), indicating that geographical factors did not significantly influence the therapeutic effect of BCIs and were not a major source of heterogeneity (Figure 3A).

**Figure 3:**
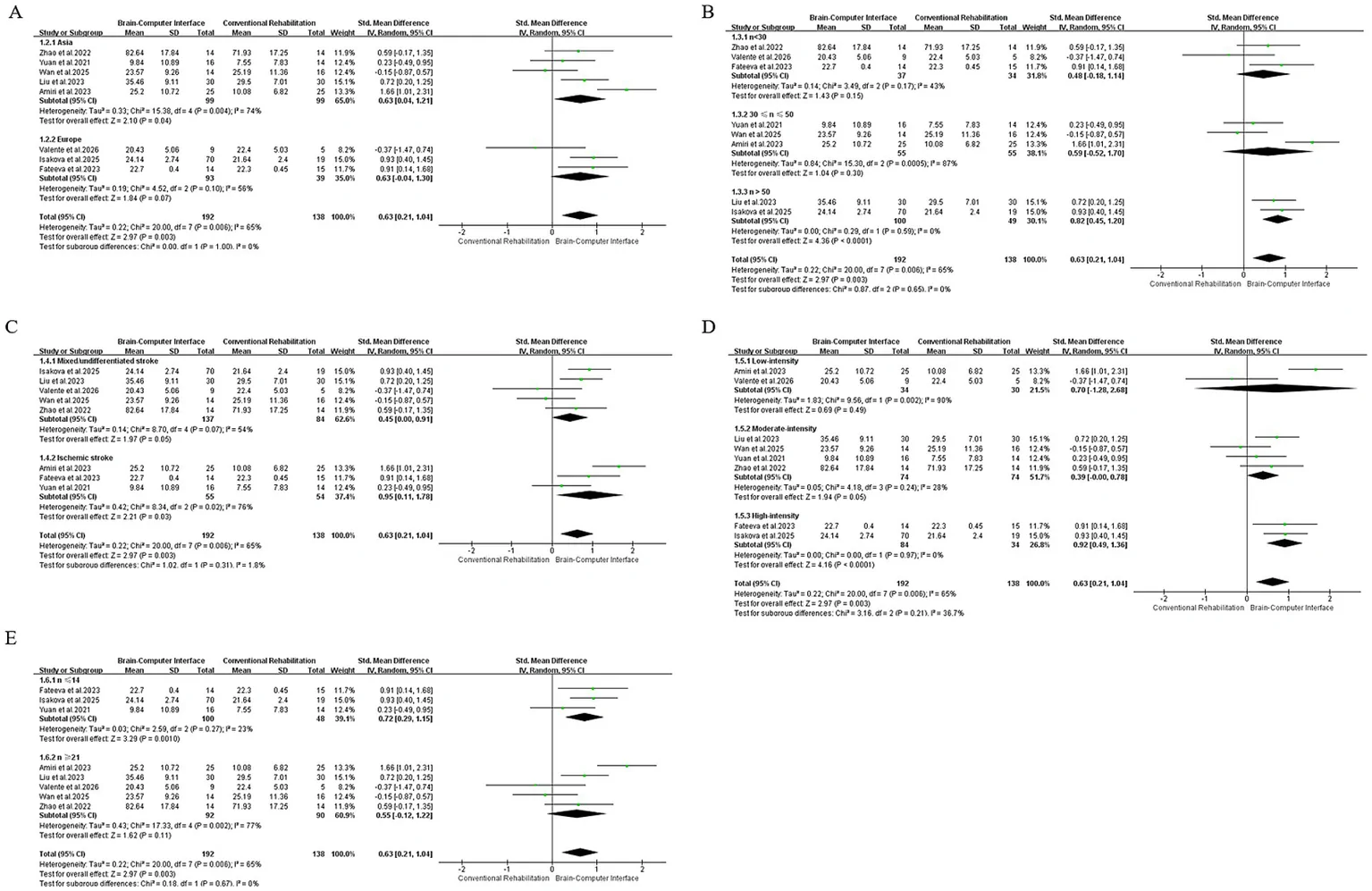
Subgroup analysis chart of the efficacy of BCI on nonmotor dysfunction in stroke patients. The figure illustrates the effects of region **(A)**, sample size **(B)**, stroke type **(C)**, intervention intensity **(D)**, and intervention duration in days **(E)** on the efficacy of BCI therapy for nonmotor dysfunction in stroke patients.

Subgroup analysis based on sample size revealed that the n < 30 subgroup included 3 studies (SMD = 0.48 [95% CI: −0.18, 1.14]), with moderate heterogeneity within the subgroup (*I*^2^ = 43%, *p* = 0.17); the 30 ≤ n ≤ 50 subgroup included 3 studies (SMD = 0.59 [95% CI: −0.52, 1.70]), with high heterogeneity within the subgroup (*I*^2^ = 87%, *p* = 0.0005); and the n > 50 subgroup included 2 studies (SMD = 0.82 [95% CI: 0.45, 1.20]), with no heterogeneity within the subgroup (I^2^ = 0.0%, *p* = 0.59). Intergroup heterogeneity tests indicated no statistically significant heterogeneity among the three subgroups (*p* = 0.65), suggesting that sample size was not a major source of heterogeneity. Additionally, the improvement effect was more stable and reliable in large-sample studies (*n* > 50) (*p* < 0.0001) (Figure 3B).

Subgroup analysis based on stroke type revealed that the mixed/undifferentiated stroke subgroup included 5 studies, with SMD = 0.45 (95% CI: 0.00, 0.91), and moderate heterogeneity was detected within the subgroup (*I*^2^ = 54%, *p* = 0.07); the ischemic stroke subgroup included 3 studies, with SMD = 0.95 (95% CI: 0.11, 1.78), and high heterogeneity was detected within the subgroup (*I*^2^ = 76%, *p* = 0.02). Intergroup heterogeneity tests revealed no statistically significant heterogeneity between the two subgroups (*p* = 0.31), suggesting that stroke type was not a major source of heterogeneity (Figure 3C).

Subgroup analysis based on intervention intensity revealed that the low-intensity subgroup included 2 studies (SMD = 0.70 [95% CI: −1.28, 2.68]), with high heterogeneity within the subgroup (*I*^2^ = 90.%, *p* = 0.002); the moderate-intensity subgroup included 4 studies (SMD = 0.39 [95% CI: −0.00, 0.78]), with low heterogeneity within the subgroup (*I*^2^ = 28%, *p* = 0.24); and the high-intensity subgroup included 2 studies (SMD = 0.92 [95% CI: 0.49, 1.36]), with no heterogeneity within the subgroup (*I*^2^ = 0.0%, *p* = 0.97). Intergroup heterogeneity tests indicated no statistically significant heterogeneity among the three subgroups (*p* = 0.21), suggesting that intervention intensity was not a major source of heterogeneity. Additionally, the effect of moderate intensity was nearly significant (*p* = 0.05), whereas the effect of high intensity significantly improved (*p* < 0.0001) (Figure 3D).

Subgroup analysis based on intervention duration revealed that the subgroup with n ≤ 14 included 3 studies (SMD = 0.72 [95% CI: 0.29, 1.15]), with low heterogeneity within the subgroup (*I*^2^ = 23%, *p* = 0.27); the subgroup with n ≥ 21 included 5 studies (SMD = 0.55 [95% CI: −0.12, 1.22]), with high heterogeneity within the subgroup (*I*^2^ = 77%, *p* = 0.002). Intergroup heterogeneity tests indicated no statistically significant heterogeneity among the two subgroups (*p* = 0.67), suggesting that intervention duration was not a major source of heterogeneity (Figure 3E).

In summary, subgroup analyses revealed variations in within-group effect estimates across different strata. However, between-subgroup differences were not significant for any of the examined factors (all *p* > 0.05; Table 3), and the moderate heterogeneity (*I*^2^ = 65%) remained largely unexplained. All subgroup findings should therefore be interpreted as exploratory.

#### 3.2.3 Impact on ADL

A total of 5 relevant studies (15, 18, 19, 21, 24) involving 317 participants demonstrated the impact of BCI-based training on ADL. The results revealed that the BCI intervention was significantly more effective than it was in the CR group (SMD = 1.18, 95% CI: 0.07, 2.29). Heterogeneity analysis revealed high heterogeneity among the included studies (*I*^2^ = 94%, *p* < 0.0001) (Figure 4A). The leave-one-out sensitivity analysis yielded SMD ranging from 0.79 (95% CI: −0.36, 1.95) after excluding Ming et al. to 2.08 (95% CI: 1.63, 2.53) after excluding Wang et al. All estimates remained positive, confirming that no single study exclusively drove the overall effect (Figure 4B). The GRADE certainty of evidence for this outcome was rated as very low (Table 2).

**Figure 4:**
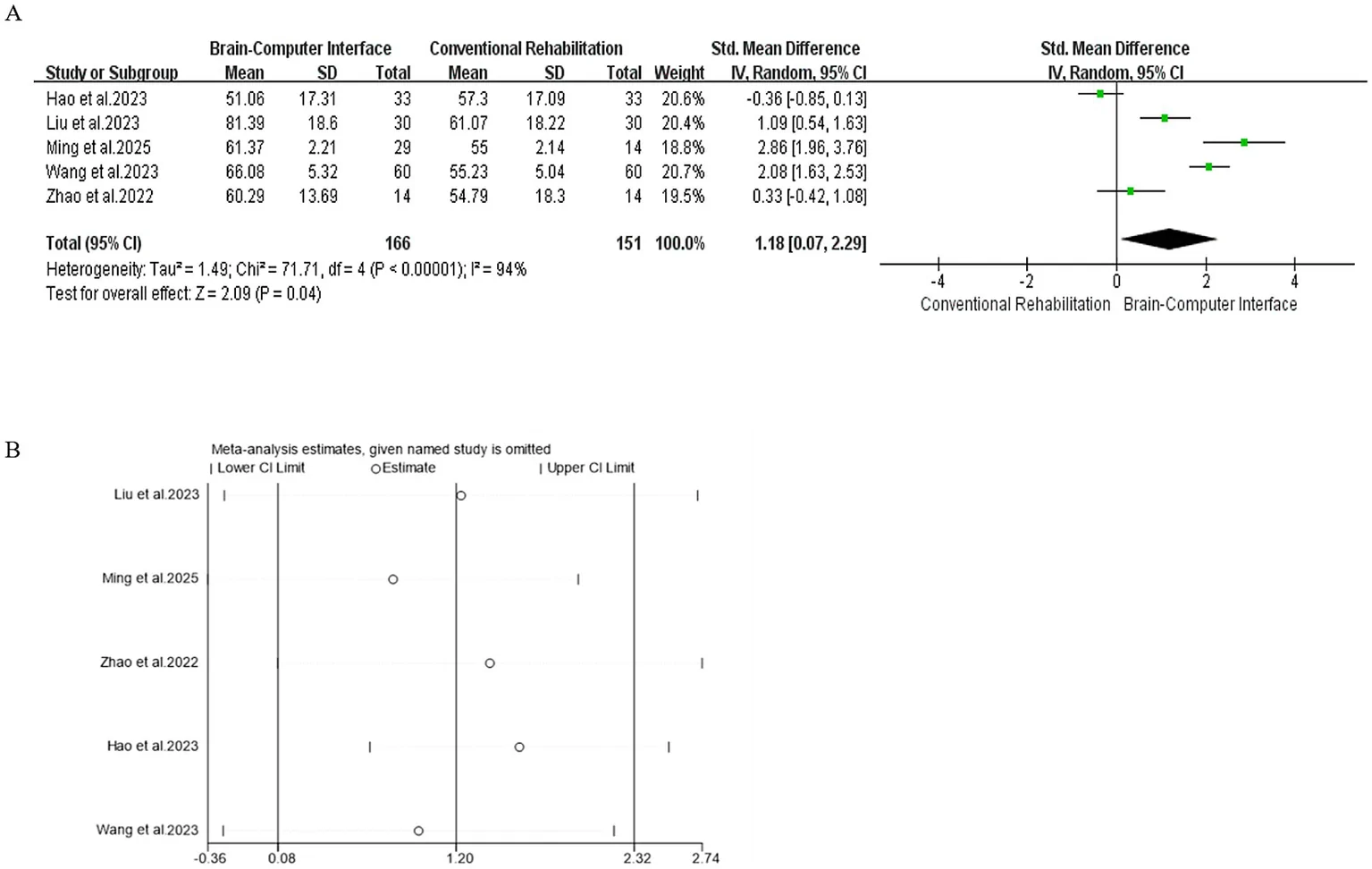
Forest plot **(A)** and sensitivity analysis plot **(B)** of the effect of the BCI on Activities of Daily Living in stroke patients.

#### 3.2.4 ADL subgroup analysis

To evaluate the robustness of the pooled results, a series of subgroup analyses were performed on the basis of sample size, whether it was the first stroke, the type of BCI, and the intervention duration (Table 4).

**Table 4:** Subgroup analysis of the effect of BCI efficacy on activities of daily living.

Grouping	Number of studies	Model	SMD	95% CI	I2	PH	*Z*	*P*	Heterogeneity between groups
Stroke									
Non-first stroke	3	Random	0.35	−0.58,1.27	87.00%	0.0005	0.74	0.460	0.0008
first stroke	2	Random	2.37	1.63,3.10	57.00%	0.130	6.31	<0.0001	
Sample									
*n* ≤ 50	2	Random	1.58	−0.90,4.06	94.00%	<0.0001	1.25	0.210	0.6600
*n*>50	3	Random	0.94	−0.51,2.39	96.00%	<0.0001	1.27	0.210	
Week									
*n* ≤ 4	3	Random	1.40	0.13,2.66	89.00%	0.0001	2.17	0.030	0.7000
*n*>4	2	Random	0.86	−1.53,3.25	98.00%	<0.0001	0.71	0.480	

Subgroup analysis based on sample size revealed that the subgroup with *n* ≤ 50 included 2 studies, SMD = 1.58 (95% CI: −0.90, 4.26), with high heterogeneity within the subgroup (*I*^2^ = 94%, *p* < 0.0001); the subgroup with n > 50 included 3 studies, SMD = 0.94 (95% CI: −0.51, 2.39), with high heterogeneity within the subgroup (*I*^2^ = 96%, *p* < 0.0001). Intergroup heterogeneity tests revealed no statistically significant heterogeneity between the two subgroups (*p* = 0.66), suggesting that sample size was not a major source of heterogeneity. (Figure 5A).

**Figure 5:**
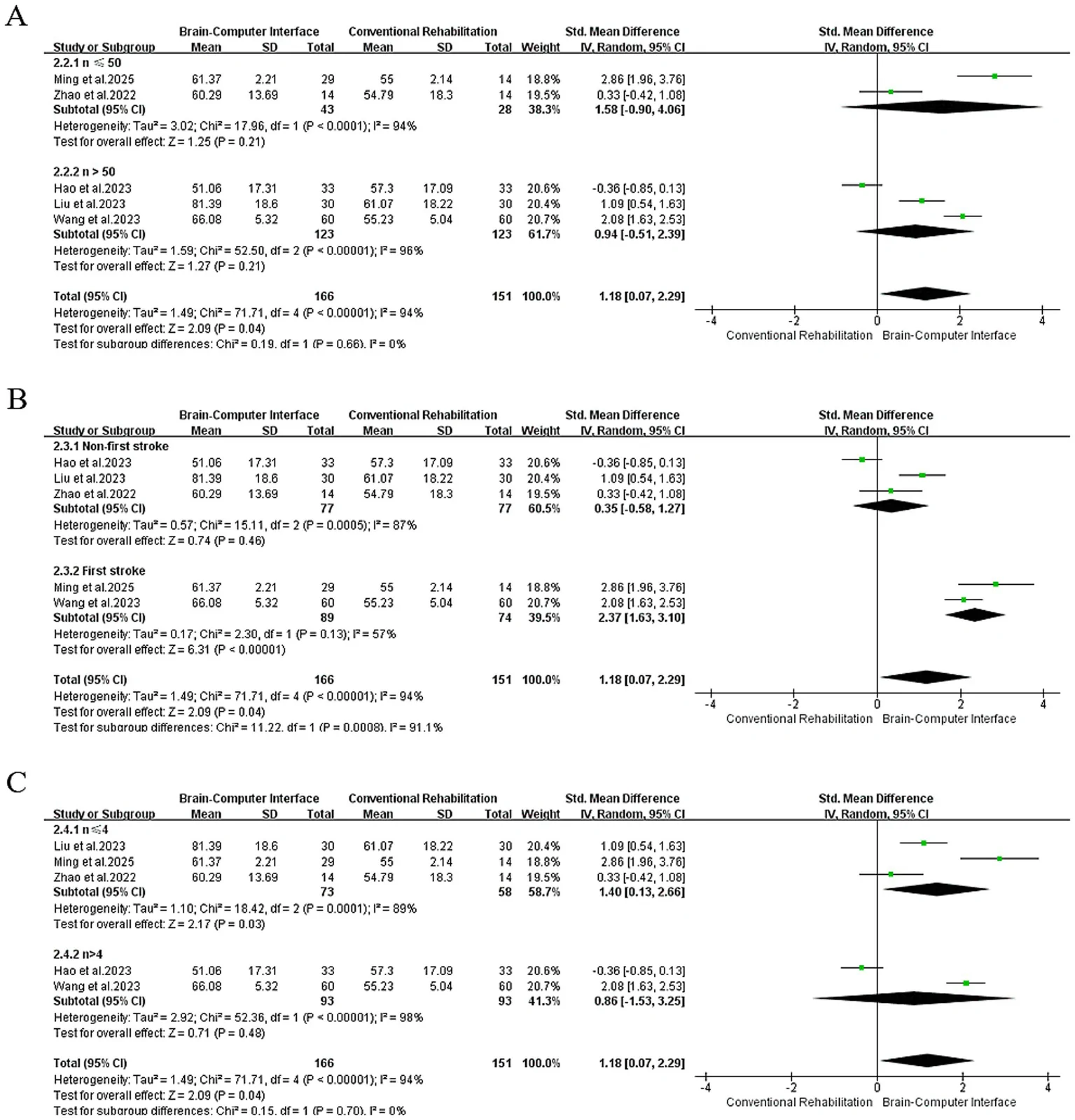
Subgroup analysis chart of the effect of the BCI on the Activities of Daily Living of stroke patients. The figure illustrates the effects of sample size **(A)**, first-ever stroke **(B)**, and intervention duration in weeks **(C)** on the efficacy of BCI therapy for ADL in stroke patients.

Subgroup analysis on the basis of whether it was the first stroke revealed that the nonfirst stroke subgroup included a total of 3 studies (SMD = 0.35; 95% CI: −0.58, 1.27), with high heterogeneity within the subgroup (*I*^2^ = 87%, *p =* 0.0005); the first stroke subgroup included a total of 2 studies (SMD = 2.37; 95% CI: 1.63, 3.10), with extremely moderate heterogeneity within the subgroup (*I*^2^ = 57%, *p* = 0.13). Intergroup heterogeneity tests revealed statistically significant heterogeneity between the two subgroups (*p* = 0.0008), indicating that whether it was the first-stroke was a major source of heterogeneity (Figure 5B). Additionally, the BCI had better effects on the rehabilitation of patients with first-onset stroke (*p* < 0.00001).

Subgroup analysis based on intervention duration revealed that the *n* ≤ 4 subgroup included 3 studies, with SMD = 1.40 (95% CI: 0.13, 2.66), and high heterogeneity was detected within the subgroup (*I*^2^ = 89%, *p =* 0.0001); the *n* > 4 subgroup included 2 studies, with SMD = 0.86 (95% CI: −1.53, 3.25), and high heterogeneity was detected within the subgroup (*I*^2^ = 98%, *p* < 0.00001). Intergroup heterogeneity tests revealed no statistically significant heterogeneity between the two subgroups (*p* = 0.70), indicating that intervention duration was not a major source of heterogeneity (Figure 5C).

Compared with conventional rehabilitation, BCI therapy significantly improved ADL. Subgroup analysis revealed that the effect was significant in first-stroke patients (*p* < 0.00001) but not in non-first-stroke patients (*p* = 0.46), with a significant between-subgroup difference (*p* = 0.0008; Table 4). These findings suggest that first-episode status may be an effect modifier, although the small number of studies limits the robustness of this conclusion. Notably, the high heterogeneity (*I*^2^ = 94%) remained largely unexplained across the subgroups.

### 3.3 Risk of publication bias

Begg’s correlation test, Egger’s linear regression test, and funnel plots were used to assess publication bias across the 8 studies included for nonmotor functions. Visually, the funnel plot showed no asymmetry in effect size distribution (Figure 6A). The results of the Begg test revealed a *p* value of 0.54 after continuity adjustment, indicating that no significant publication bias was detected (Table 5, Figure 6B). Egger’s regression analysis revealed a bias of 2.750 with no statistical significance (*p* = 0.214), further confirming that there was no evident publication bias (Table 5, Figure 6C) and suggesting that no significant publication bias was detected by the available tests.

**Figure 6:**
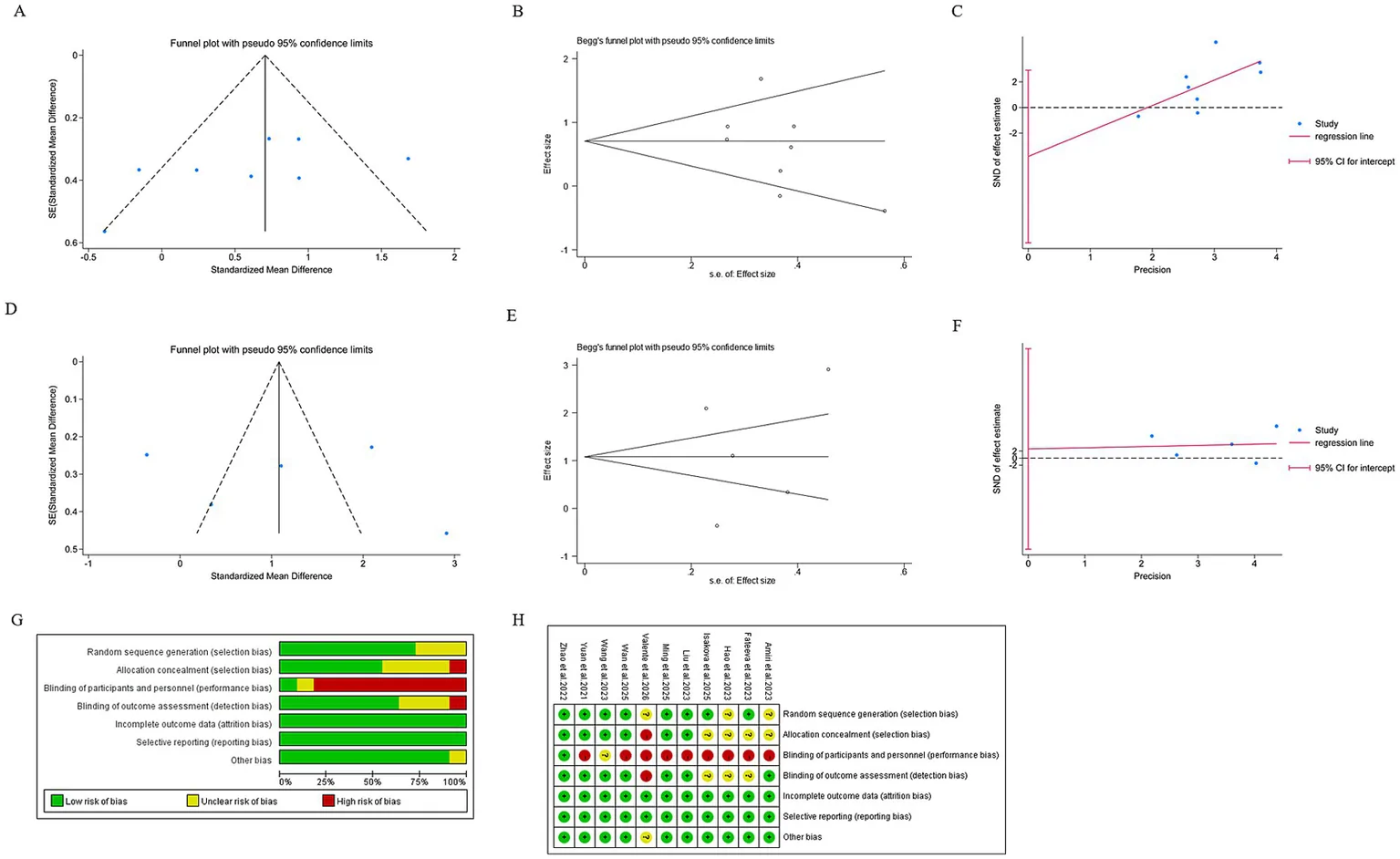
The risk of publication bias for the included literature. The figure presents the funnel plot of studies included for nonmotor dysfunction **(A)**, Begg’s test plot **(B)**, and Egger’s test plot **(C)**; the funnel plot of studies included for Activities of Daily Living **(D)**, Begg’s test plot **(E)**, and Egger’s test plot **(F)**; and the bias risk map **(G)** and summary table of bias risks **(H)** for all studies included in this study.

**Table 5:** Publication bias tests for non-motor function outcomes.

Test	Statistics	Statistical value	*t/z*	*P*	95% CI	Number of studies
Begg’ test	Kendall’s score(P-Q)	−6	—	—	—	8
	Std. Dev.of score	8.08	—	—	—	
	Uncorrected Z value	—	−0.74	0.62	—	
	Corrected Z value	—	0.46	0.54	—	
Egger’s test	slope	0.9419148	2.11	0.079	−0.3175106,4.292055	8
	Bias	2.750237	−1.39	0.214	−10.55324,2.905933	

Begg’s correlation test, Egger’s linear regression test, and funnel plots were used to assess publication bias for the 5 studies included in the ADL. Visually, the funnel plot revealed no asymmetry in the distribution of effect sizes (Figure 6D). Begg’s test (*p* = 1.00) and Egger’s regression (bias = 9.066, *p* = 0.790) similarly indicated no significant publication bias (Table 6, Figures 6E,F). However, given the limited number of studies (fewer than 10), these results should be interpreted with caution and do not definitively exclude the possibility of publication bias.

**Table 6:** Publication bias tests for activities of daily living outcomes.

Test	Statistics	Statistical value	t/z	P	95% CI	Number of Studies
Begg’ test	Kendall’s score(P-Q)	0	—	—	—	5
	Std. Dev.of score	4.08	—	—	—	
	Uncorrected Z value	—	−0.24	0.81		
	Corrected Z value	—	1.00	1.00	—	
Egger’s test	slope	2.614408	0.13	0.904	−7.975721,8.664706	5
	bias	9.065521	0.29	0.790	−26.21812,31.48295	

Additionally, the Cochrane Risk of Bias Assessment Tool was used to evaluate the methodological quality of all included studies. The results revealed that the overall methodological quality of the included studies was good, with most having a low risk of bias. However, in terms of performance bias, only a few studies had a low risk, while most had a high risk, which is a common issue in research in this field, as BCI interventions cannot blind participants and implementers (Figures 6G,H).

## 4 Discussion

This study systematically evaluated the current evidence the effects of BCI technology on nonmotor functions (primarily cognitive function) and ADL in stroke patients through a systematic review and meta-analysis. The integrated results suggested that BCI training may be beneficial for improving nonmotor functions and ADL in stroke patients. It should be noted that four of the five studies pooled for the ADL outcome used the MBI, which measures functional independence, whereas only one used the WHOQOL-BREF (Wang et al.). Due to the limited number of studies, separate analysis was not feasible. Thus, the pooled results should be interpreted primarily as an effect on ADL, with caution when generalizing to health-related quality of life. However, these results exhibited high heterogeneity, which remained largely unexplained despite extensive subgroup analyses.

Despite extensive subgroup analyses, the high heterogeneity (nonmotor: *I*^2^ = 66.3%; ADL: *I*^2^ = 94.5%) remained largely unexplained, as none of the examined factors showed significant intergroup differences (all *p* values > 0.05). Potential clinical sources include variations in control interventions, unreported baseline cognitive status, variable follow-up durations, and heterogeneous BCI protocols (feedback modalities, training paradigms). These factors underscore the need for standardized, multicenter RCTs with consistent reporting of clinical details.

Although our quantitative synthesis focused on cognitive function, emerging evidence suggests that BCI may hold potential across other nonmotor domains. With respect to emotional regulation, Ma et al. systematically reviewed the role of the BCI in monitoring and regulating poststroke emotional disorders via closed-loop neurofeedback systems (11), and Hu et al. reported that EEG-based neurofeedback significantly reduced depression and anxiety in randomized controlled trials (26). For speech rehabilitation, BCI-based neurofeedback and P300 paradigms have been identified as viable therapeutic alternatives in aphasic patients, although evidence remains limited to small-sample studies (27). With respect to swallowing dysfunction, motor imagery-based BCIs (MI-BCIs) can activate swallowing-related brain regions and have therapeutic potential, but standardized protocols are lacking, and issues such as signal decoding difficulties and significant individual differences limit clinical application (28). Overall, current evidence in these domains remains preliminary, with most studies being small-sample feasibility or proof-of-concept investigations. Well-designed, large-scale RCTs are urgently needed before clinical recommendations can be made.

This study has several limitations. First, the relatively limited number of studies (8 for nonmotor function and 5 for ADL) may affect the stability of the results. Second, most included studies had a high risk of performance bias because of the inability to blind participants and therapists—a field-wide limitation of BCI research—which may introduce expectancy effects and partially contribute to the observed heterogeneity. Third, the cognitive outcome measures varied across studies (MoCA, SDMT, and LOCTA), limiting comparability and failing to fully capture the multidimensional nature of cognitive function. Fourth, the number of included studies was insufficient for reliable meta-regression analysis (a minimum of 10 studies is recommended). Fifth, most of the included studies did not provide sufficient technical details on signal acquisition, feature extraction, or classification algorithms (Supplementary Table S1), limiting comparability across interventions. Sixth, one of the included studies [Amiri et al. (15)] utilized RehaCom, a computer-assisted cognitive training program, rather than a conventional EEG-based BCI, despite its keywords referencing brain–computer interfaces. We addressed this by performing a sensitivity analysis excluding this study, which confirmed that our primary findings remained robust (Section 3.2.1), and retained the study to reflect the heterogeneity in BCI terminology across the literature. Seventh, the PROSPERO protocol was retrospectively registered (July 17, 2026), and no subgroup analyses were pre-specified; all subgroup analyses reported in this study are exploratory and should be interpreted as hypothesis-generating. Future BCI studies should adopt standardized technical reporting and consistent clinical outcome reporting to enable more refined analyses.

## 5 Conclusion

Current evidence suggests that BCI-based training shows preliminary promise for improving cognitive function and ADL in stroke patients, although the overall certainty remains limited. In clinical practice, BCI may serve as a complementary tool to conventional rehabilitation but should not replace standard therapies. For research, future studies should prioritize the standardization of assessment tools, the optimization of intervention protocols, and the identification of patient subgroups most likely to benefit. The successful translation of BCI from research to practice will require more rigorous, large-scale, and long-term evidence.

## Data Availability

The raw data supporting the conclusions of this article will be made available by the authors, without undue reservation.
