# Supplementary material for: The application of brain-computer interface on cognitive function and activities of daily living in stroke patients: a systematic review and meta-analysis
Source: Front Neurol. 2026 Oct 19;17:1862193. doi: 10.3389/fneur.2026.1862193 (PMC13725544; doi:10.3389/fneur.2026.1862193)
Supplement: Supplementary file 1 [file Table_1.docx]

Supplementary Table S1. BCI technical specifications of the included studies

| **Study** | **Signal Acquisition** | **Electrode Placement** | **Feature Extraction** | **Classification** | **Feedback** |
| --- | --- | --- | --- | --- | --- |
| Liu et al. 2023[15] | EEG (MI-BCI) | 14 channels | Not reported | Not reported | Visual + FES |
| Wan et al. 2025[16] | EEG (MI-VR-BCI) | Prefrontal cortex | Alpha/Beta energy ratio | Attention index | VR + visual |
| Isakova et al. 2025[17] | EEG (P300/mu-rhythm) | Not reported | P300 ERP/mu pattern | Not reported | Biofeedback |
| Ming et al. 2025[18] | EEG (KI-BCI) | Fp1,Fp2,F3,F4,C3,C4,Fz,Cz | MRCP + DTW | Threshold-based | Visual+auditory+robotic |
| Zhao et al. 2022[19] | EEG (SSMVEP) | O1,O2,Oz,PO3,PO4,POz | SSMVEP (Newton's ring) | Frequency-specific | Visual (robot) |
| Fateeva et al. 2023[20] | EEG (P300 speller) | 8 leads | P300 ERP | Accuracy index | Visual |
| Hao et al. 2023[21] | EEG (BCI-RTS-FES) | Not reported | Not reported | Not reported | FES |
| Yuan et al. 2021[22] | EEG (BCI-PT) | Single-channel Fp1 | Beta/Alpha ratio + FFT | Attention index | Visual+auditory+somatosensory |
| Amiri et al. 2023[23] | Non-EEG (RehaCom) | N/A | N/A | N/A | Computerized |
| Wang et al. 2025[24] | EEG-BCI | Prefrontal motor regions | Feature extraction | Pattern recognition (>77.5%) | Visual + FES |
| Valente et al. 2026[25] | EEG (VR-MI-BCI) | 32 channels (10-10) | Individualized ERD + CSP | LDA | VR + vibrotactile |

Abbreviations: FES, functional electrical stimulation; VR, virtual reality; MI, motor imagery; SSMVEP, steady-state motion visual evoked potential; MRCP, movement-related cortical potential; DTW, dynamic time warping; ERP, event-related potential; CSP, common spatial pattern; LDA, linear discriminant analysis; FFT, fast Fourier transform.
